# Purpose reflection benefits minoritized students’ motivation and well-being in STEM

**DOI:** 10.1038/s41598-023-50302-1

**Published:** 2024-01-03

**Authors:** Amanda B. Diekman, Mansi P. Joshi, Andrew D. White, Quang-Anh Ngo Tran, Jayshree Seth

**Affiliations:** 1 Department of Psychological and Brain Sciences, Indiana University, Bloomington, USA; 2 Present Address: Veris Insights, Washington, USA; 3 3M Company, Minnesota, USA

**Keywords:** Psychology, Human behaviour

## Abstract

Students from groups historically excluded from STEM face heightened challenges to thriving and advancing in STEM. Prompting students to reflect on these challenges in light of their purpose can yield benefits by helping students see how their STEM work connects to fundamental motives. We conducted a randomized, controlled trial to test potential benefits of reflecting on purpose—their “why” for pursuing their degrees. This multimethod study included 466 STEM students (232 women; 237 Black/Latinx/Native students). Participants wrote about their challenges in STEM, with half randomly assigned to consider these in light of their purpose. Purpose reflection fostered benefits to beliefs and attitudes about the major, authentic belonging, and stress appraisals. Effects were robust across race and gender identities or larger for minoritized students. Structural and cultural shifts to recognize students’ purpose in STEM can provide a clearer pathway for students to advance.

## Introduction

Pursuing a degree in science, technology, engineering, and mathematics (STEM) is a challenging journey: High-achieving students learn greater quantities of material more quickly and with greater independence than ever before. Further, the terrain is more challenging for students from groups historically excluded from STEM: In physical sciences and engineering in particular, women and Black, Latinx, and Native students continue to be underrepresented relative to the US population^1^. Students from historically excluded groups can experience their courses, and indeed the broader fields of science and engineering, differently. All students who meet with challenging material can question whether this path is the right one for them, but for students minoritized by race or gender, these doubts compound for several reasons. They may not see similar others modelling the path; they lack structural resources to buffer uncertainties; and they contend with experienced and potential bias. Local STEM departments can operate on cultural defaults that explicitly and implicitly exclude minoritized students (e.g., masculine defaults^2^). Yet it is imperative for a vital STEM workforce that we ensure that pathways to STEM careers are open and accessible to a broad range of students.

The current research provides empirical evidence for a route to improving the quantity and quality of the STEM workforce by prompting STEM students to consider their challenges in light of their own *purpose*—that is, their “why” for pursuing their degrees. We join practitioners in science and engineering who call for early career scientists to persevere to find their purpose^3^. Our theoretical approach to question emerges from *goal congruity theory*^4,5^, which posits that preferred pathways that those that are seen as fulfilling highly valued goals. Across multiple contexts, evidence supports the goal congruity principle that beneficial cognitions and attitudes result from contexts that signal *goal affordances*, or opportunities to fulfill fundamental motives. Here, we investigate whether articulating purpose elevates these goal affordances: Does articulating their “why” bolster student perceptions that STEM fulfills their most important values? This investigation matters because the “why” is not always clear for students, and even if it is, they may not perceive their purpose supported in their STEM courses and majors. Yet there is ample reason to suspect that seeing STEM as aligning with purpose will motivate and encourage students—perhaps especially students from historically excluded groups.

### Articulating purpose: fulfilling agentic and communal motives

When students consider their own purpose in pursuing STEM, they are likely to bring to the forefront of their minds commonly expressed values along dimensions of *agency* and *communality*. *Agency* includes self-oriented aspects such as achievement, mastery, status, or influence; *communality* includes other-oriented aspects such as altruism, collaboration, connection to others, and serving a broader community^6,7^. Both values contribute to optimal functioning^8,9^, but the emphasis placed on them can vary across individuals and contexts^10^. The fundamental principles of goal congruity theory suggest that seeing agentic and communal values as possible in STEM is essential for motivation and persistence.

To see whether agentic and communal values emerge among STEM professionals, we can examine reported inspirations for pursuing a STEM career. As shown in Table 1, individual-focused reasons (being passionate or having a career in a respected field) were more often cited by White men, whereas reasons focused on social impact (solving world’s biggest challenges or making a difference) were especially cited by White women and Black/Hispanic STEM professionals.

**Table 1:**
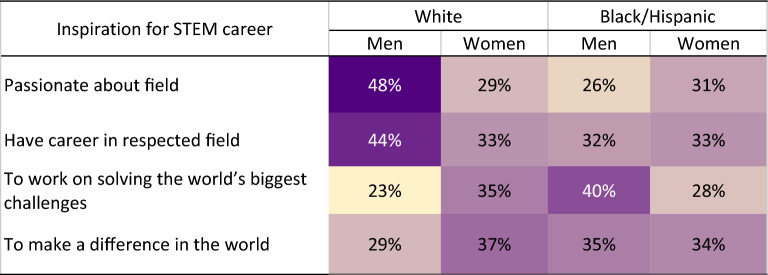
STEM professionals’ inspiration for pursuing STEM career by race and gender (State of Science Index).

Unpublished US subsample survey data retrieved from the State of Science Index (3M, 2022). Participants responded to the question “What is your inspiration for pursuing a STEM career?” and selected from a range of options.

Notably, the inspirations that STEM professionals reported varied by both gender and race. Yet the modal STEM department may not prioritize the communally-oriented reasons emphasized by minoritized groups. Common beliefs hold that STEM fields lack opportunities to fulfill communal goals^11^ or to work with communally-oriented faculty^12^. Further, the cultures in engineering and physical sciences departments can particularly emphasize agency over communality^10^. The challenge thus is to build clearer bridges between STEM educational activities and the purposes articulated by students.

Because STEM departments can be seen by students as operating at a communality deficit, highlighting the ways that STEM fields can fulfill prosocial goals holds particular power^4,5,13^. Highlighting communal opportunity in STEM fosters greater motivation^14^ and better performance^15^. When students (even outside of the STEM pathway) reflect on *why* scientists do their work, students generate more communally-oriented content^16^. Considering “why” might bring to mind how science and engineering contribute to communal purpose. Sometimes the beneficial “why” is quite concrete and practical: Prompting students to consider the utility value of their science or math coursework increases student motivation and performance^17^. At other times, the “why” is more abstract: Prompting students to consider the self-transcendent purpose of their coursework yielded greater persistence and graduation rates for first-generation students^18^.

If articulating purpose taps into fundamental motives, similar benefits might emerge across racial identities, because evidence suggests that agency and communality are highly valued within and across racial groups. For example, Afrocentric values center communally-oriented ideals related to community, collaboration, and justice^19,20^. Yet group membership likely shapes the experience and endorsement of fundamental motives. For instance, the facets of communal purpose might diverge for minoritized and majoritized groups. McGee and colleagues^13,21^ advocate that STEM faculty incorporate an *equity ethic* into their material to more fully engage Black and Latinx students. If STEM courses and communicators can clarify these paths, both students and the science will reap the benefit.

### A fuller picture of experience: authentic belonging and stress appraisals

Integrating purpose into STEM work can bolster students’ belonging and resilience through goal congruity processes. Students’ clarity about how their STEM path aligns with their fundamental motives can contribute to a sense that one’s true self is seen and valued within STEM. Prior goal congruity evidence shows that students (especially women) who wrote about science as including communal opportunities reported greater belonging in science^22^. This sense of purpose and goal alignment can especially be valuable as students meet with challenges; otherwise, struggle can be a sign that the student should exit (particularly for students contending with identity threats). Individuals who experience contexts as aligning with their values tend to experience authenticity in those contexts^23^; such experiences of authenticity can matter especially when students occupy identity-threatening contexts^24^.

Articulating purpose in STEM also holds potential to reduce deleterious psychological stress responses. Psychological stress occurs when the demands of stressors surpass efficacy to cope with them or the resources for coping^25^. In contrast, more adaptive responses occur when individuals believe that their capacities surpass their stressors. Students minoritized by their race, gender, or multiple identities experience stressors related to challenging work, as well as additional stressors due to potential bias, resource disparities, and isolation or exclusion. A novel contribution of the current research is to expand goal congruity evidence to stress appraisals: Does articulating purpose lead to viewing the STEM pathway as affording communal and agentic goals, and are these in turn associated with reduced stress appraisals? This investigation offers an opportunity to integrate evidence that perceived autonomy, competence, and relatedness predict well-being^26^, that social connect buffers against stress and illness^27^, and that perceived control matters for well-being^28^. Students’ ability to see their challenges in STEM in light of their purpose can cue their perceptions of agentic and communal affordances, and these in turn can set the stage for more resilient stress appraisals.

### Current research

We conducted a randomized, controlled trial testing the benefits of a brief elicitation of students’ purpose in STEM. Our goal was to determine what motivational and attitudinal benefits arise from reflecting on challenges along with purpose, and whether these effects emerge across gender and race/ethnicity. This research examined three central questions:

1. does articulating purpose benefit students, and are these benefits observed among groups historically excluded from STEM?
2. are the benefits of purpose reflection on authentic belonging and stress appraisals related to perceived opportunities to fulfill communal and agentic motives in STEM?
3. Do the challenges and purposes articulated by STEM students vary across gender and race/ethnicity?

To answer these questions, we studied a sample of 466 students in STEM (232 women and 234 men), with sufficient numbers to examine responses of identities well-represented in STEM (White/Asian) and minoritized in STEM (Black/Latinx/Native; see NSF, National Science Board, 2022). Students identified their race/ethnicities as White (n = 280; 60.1%), Latino(a/x) (n = 134; 28.8%), Black/African American students (n = 93; 20%), Asian/Asian American (n = 11; 2.4%); and Native American/Indigenous students (n = 10; 2.1%). Post hoc sensitivity power analyses indicated a detectable effect size of *f* = 0.130 for a 2 (Reflection Condition) × 2 (Gender) × 2 (Majoritized or Minoritized Race/Ethnicity) between-subjects analysis of variance (ANOVA), power = 0.80; α = 0.05, *n* = 466;^29,30^.

In the brief online exercise, each student reflected on challenges in their STEM major. The presence of purpose constituted the experimental manipulation: Half of the participants were randomly assigned to consider these challenges integrated with their purpose. Students wrote for 5 min following their randomly-assigned instruction, and then completed self-report assessments (see "Method").

## Results

Key analyses were 2 Reflection Condition (challenge or purpose/challenge) × 2 Racial Status (minoritized, majoritized) × 2 Gender (women, men) between-subjects ANOVAs testing all main effects and interactions. We present all significant effects of reflection condition and interactions between reflection and race and/or gender; however, student identities did not systematically moderate the effect of purpose reflection. All other significant effects (e.g., main effects of race and gender) are reported in Supplementary Materials. In brief, those findings reveal greater difficulties for students identifying as women, as Black/Latinx/Native, or both—they report less beneficial beliefs and attitudes about the major, less authentic belonging, and greater stress appraisals.

With this context in mind, we turn to examining whether the brief reflection of purpose bolsters students from historically excluded groups in STEM. As intended, the experimental manipulation significantly influenced written responses. Purpose themes more frequently appeared among participants directed to write about their purpose, whereas challenge themes more frequently appeared among participants directed to focus only on challenges (chi-square *p*s =  < 0.001-0.024). Purpose themes were rarely spontaneously generated in the challenge condition.

### Research question 1: Does reflecting on purpose benefit both majoritized and minoritized students?

#### Effects of purpose integration on major/career beliefs and attitudes

**Figure 1:**
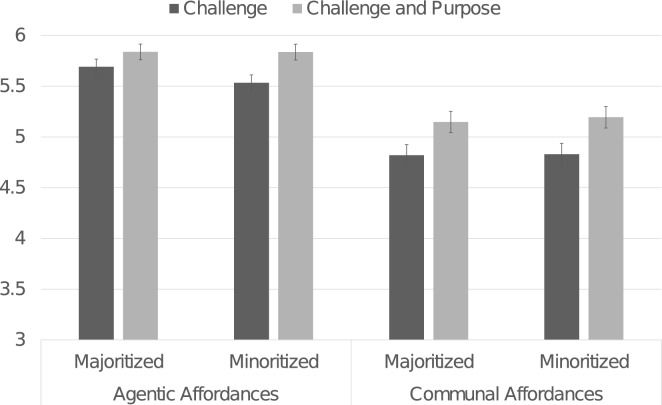
Effects of purpose reflection on perceived goal affordances in the major. *Note.* Ratings ranged from 1 to 7, with higher numbers reflecting more of the construct. Error bars present ± 1 standard error.

Reflecting on challenges integrated with purpose, relative to reflecting on challenges alone, increased students’ belief that their STEM major offered opportunities to meet their goals, *F*(1, 458) = 12.55, *p* < 0.001, *η*_*p*_^2^ = 0.027. The purpose benefit occurred for both communal and agentic affordances (see Fig. 1). The benefit was stronger for men than women, Purpose Reflection × Gender interaction: *F*(1, 458) = 3.867, *p* = 0.050, *η*_*p*_^2^ = 0.008.

**Figure 2:**
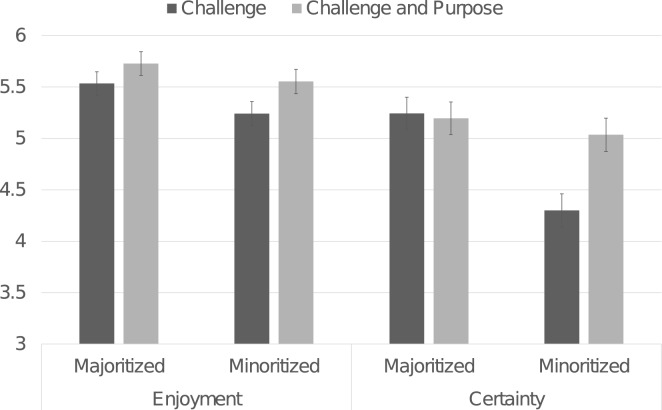
Effects of purpose reflection on attitudes about the major. *Note.* Ratings ranged from 1 to 7, with higher numbers reflecting more of the construct. Error bars present ± 1 standard error.

As shown in Fig. 2, students reflecting on purpose reported greater enjoyment in pursuing the STEM major, *F*(1, 457) = 4.71, *p* = 0.031, *η*_*p*_^*2*^ = 0.010, *d* = 0.21, and greater certainty in pursuing the STEM major, *F*(1, 457) = 4.612, *p* = 0.032, *η*_*p*_^*2*^ = 0.010, *d* = 0.19. Further, purpose reflection especially elevated minoritized students’ certainty about the major: Purpose Reflection × Race interaction, *F*(1, 457) = 5.967, *p* = 0.015, *η*_*p*_^*2*^ = 0.013. Purpose reflection increased major certainty for racially minoritized students, *F*(1, 457) = 10.322, *p* = 0.001, *η*_*p*_^*2*^ = 0.022, *d* = 0.37 (challenge: *M* = 4.33; *SD* = 1.87; purpose: *M* = 5.03, *SD* = 1.75). Majoritized students’ certainty was stably high across condition (challenge: *M* = 5.22, *SD* = 1.72; purpose: *M* = 5.26, *SD* = 1.65), *F*(1, 457) = 0.045, *p* = 0.833, *η*_*p*_^*2*^ < 0.001, *d* = 0.02.

#### Effects of purpose integration on student psychological experience

**Figure 3:**
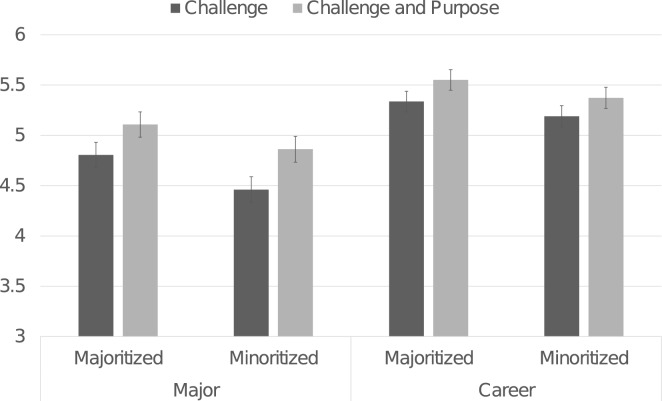
Effects of purpose reflection on authentic belonging in the major and anticipated career. *Note.* Ratings ranged from 1 to 7, with higher numbers indicating greater amounts of the construct. Error bars present ± 1 standard error.

Students who reflected on their purpose reported more positive psychological experiences across multiple measures. As shown in Fig. 3, students who reflected on purpose, relative to the challenge, reported greater authentic belonging in the major, *F*(1, 458) = 7.68, *p* = 0.006,* η*_*p*_^*2*^ = 0.016, *d* = 0.24, and anticipated descriptively more authentic belonging in their future careers, *F*(1, 458) = 3.686, *p* = 0.055,* η*_*p*_^*2*^ = 0.008, *d* = 0.18.

Further, students who reflected on purpose, relative to challenge, reported reduced stress appraisals (which reflect that reported stress outweighs capacity to handle stress), *F*(1, 458) = 19.26, *p* < 0.001,* η*_*p*_^*2*^ = 0.040. Students who wrote about their challenges reported greater stress than capacity (*M* = 0.61, *SD* = 2.01), whereas students who reflected on purpose reported greater capacity than stress *(M* = -0.19, *SD* = 2.07*)*.

**Figure 4:**
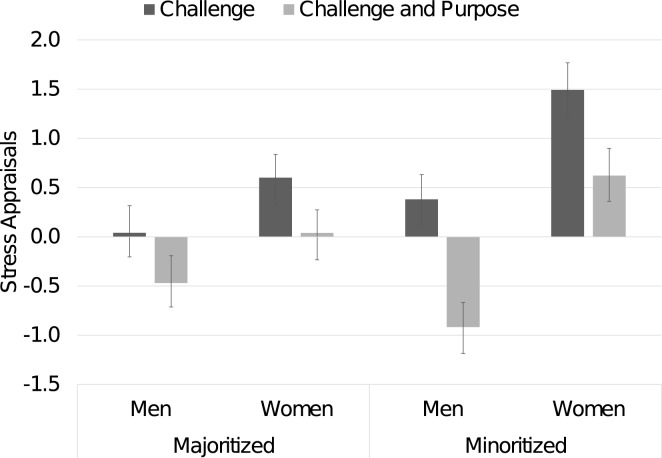
Effects of purpose reflection on stress appraisals. *Note.* Positive stress appraisal scores denote more stress than capacity to handle stress; negative scores denote less stress than capacity to handle stress. Error bars present ± 1 standard error.

Given the elevated stress appraisals reported by minoritized women in particular (see Supplement for details), we examined variation across identity groups (see Fig. 4). We note that the purpose reflection effect was not significantly moderated by identity groups: Reflection Condition × Race interaction,* F*(1, 458) = 2.205, *p* = 0.138, *η*_*p*_^*2*^ = 0.005; three-way Reflection Condition × Gender × Race, *F*(1, 458) = 0.425, *p* = 0.515, *η*_*p*_^*2*^ = 0.001. Although reflecting on purpose reduced stress appraisals for racially minoritized women, this group remained at the highest levels of stress appraisal after reflection. Further, contrasts showed a significant purpose benefit for minoritized men, *d* = 0.59, *p* < 0.001, and minoritized women, *d* = 0.45, *p* = 0.023, but smaller and nonsignificant benefits for majoritized men, *d* = 0.26, *p* = 0.168, and majoritized women, *d* = 0.27, *p* = 0.119.

### Research question 2: Are effects of purpose reflection on belonging and stress appraisal mediated by perceived opportunities to fulfill agentic or communal goals?

**Figure 5:**
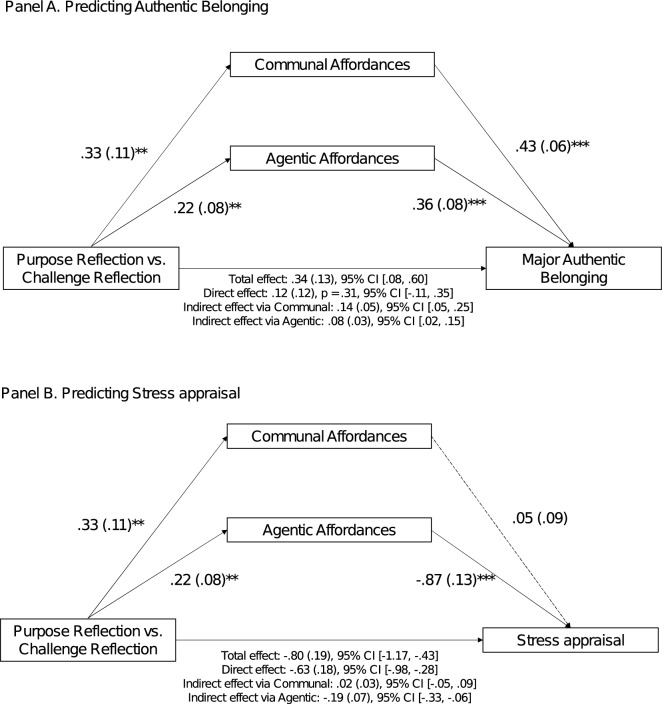
Indirect effects of purpose reflection on student experience via perceived affordances. *Note.* Models were tested with communal and agentic affordances as parallel mediators using Hayes^39^ PROCESS, model 4, with 5000 bootstrap samples. Reflection condition was dummy-coded as 1 = purpose, 0 = challenge. Betas are unstandardized. Standard errors are in parentheses. *p < .05; **p < .01; ***p < .001.

We then tested possible psychological pathways for the effects of purpose reflection on the theoretically-relevant student experience variables of belonging and stress appraisals (see Fig. 5). For authentic belonging, both agentic and communal affordances mediate the effect (see Panel A); in contrast, for reduced stress appraisals, only agentic affordances mediate (see Panel B). For students in the STEM pathway, articulating their purpose increased their belief that they can meet their self-oriented and other-oriented goals in their STEM majors; these perceived goal opportunities were distinctly associated with beneficial outcomes.

### Research question 3: Do challenges and purposes vary across student race and gender?

We employed thematic coding to document the content of students’ writing about their challenges and purposes.

#### Challenge condition

Table 2 depicts that the most frequently mentioned challenges were commonly held across groups: Coursework difficulties (*n* = 173), social stressors (*n* = 52), and uncertainty about their major or career (*n* = 48).

**Table 2:** Characterizing challenge themes: frequencies by race and gender.

Challenge theme	Minoritized					Majoritized				
	Women		Men		Overall	Women		Men		Overall
	n = 51		n = 62		*n = 113*	n = 70		n = 51		*n = 121*
	*n*	prop	*n*	prop	*n*	*n*	prop	*n*	prop	*N*
Coursework	30	.59	39	.63	*69*	57	.81	47	.92	*104*
Social Stressors	16	.31	10	.16	*26*	17	.24	9	.18	*26*
Uncertainty	13	.25	14	.23	*27*	13	.19	8	.16	*21*
Work & Family	7	.14	13	.21	*20*	11	.16	5	.10	*16*
Identity	9	.18	2	.03	*11*	10	.14	0	.00	*10*
Resources	1	.02	7	.11	*8*	6	.09	1	.02	*7*

Data for participants (N = 234) in the challenge condition. Themes are listed in descending frequency across the whole sample. Proportions (prop.) are calculated within group and do not sum to 100% within column because participants could report multiple challenges.

Chi-square goodness of fit tests performed on each theme detected no significant differences in the proportions of each identity group mentioning challenge themes (*p*s = 0.165–0.676). Examination across themes, however, shows that the dispersion of challenges varied by majoritized vs. minoritized status: Minoritized students report challenges across themes, whereas majoritized students concentrate in coursework (i.e., more than 80% of majoritized vs. approximately 60% of minoritized students). In parallel, identity stressor themes emerge among women and especially among racially minoritized women. Identity stressors included both overt prejudice as well as contending with the white male “default”^2^ in STEM.

“My major is difficult because the technology involved with audio engineering changes frequently, as well as the culture. It is also a very male-dominated industry, and as a first generation collegiate female, I have a hard time feeling like I fit in. I have yet to face any explicit sexism or discrimination, but it is always subliminally present. I have mostly male professors, and it is clear they just have different classroom expectations that I, as a girl, have been taught to adhere to. For me, it is be quiet and polite. Sit still, pay attention, raise your hand to ask questions, take diligent notes. But in many of my classes, the guys yell out answers, crack jokes that everyone laughs at, speak over each other and myself. It is just a different culture, and I don't think the male students or professors see how that is inherently creating an environment that women are not familiar with or welcome in.” (White woman; audio engineering).

Some students faced multiple, compounding challenges, such as identity stressors, coursework difficulties, and scarce resources.

“I am low income and so to survive I have to spend a lot of my time working non skilled labor just to make ends meet. It leaves little time for studying and pursuing my passions of Computer Science … If I spend more time studying, then I won't have enough money to pay for my basic needs such as food, shelter, and clothing … Being a first generation college student makes it really tough to know what the right step is and what to do next when it comes to my education because I don't have anyone in my family or close social circle to guide me since I'm the first one to walk this path.” (Latino man; Computer Science).

Coursework challenges are paramount for all students, but students from historically excluded groups must navigate these alongside a range of other ongoing obstacles.

#### Purpose reflection condition

Students asked to reflect on their purpose most frequently mentioned themes relating to competence (*n* = 183), prosociality (*n* = 104), and financial rewards (*n* = 67; see Table 3).

**Table 3:** Characterizing purpose themes: frequencies by race and gender.

Purpose theme	Minoritized					Majoritized				
	Women		Men		Overall	Women		Men		Overall
	n = 58		n = 55		*n = 113*	n = 53		n = 66		*n = 119*
	*n*	prop	*n*	prop	*n*	*n*	prop	*n*	prop	*n*
Competence	37	.64	42	.76	*79*	46	.87	58	.88	*104*
Prosocial	28	.48	21	.38	*49*	27	.51	28	.42	*55*
Financial	13	.22	14	.25	*27*	15	.28	25	.38	*40*
Representation	16	.28	0	.00	*16*	4	.08	0	.00	*4*
Status	9	.16	4	.07	*13*	6	.11	4	.06	*10*
Connection	8	.14	1	.02	*9*	5	.09	2	.03	*7*

Data for participants (N = 232) in the purpose condition. Themes are listed in descending frequency across the whole sample. Proportions (prop.) are calculated within group and do not sum to 1.0 within column because participants could report multiple purposes.

Chi-square goodness-of-fit tests within each theme detected no significant effects of identity group (*p*s = 0.41–0.75). Again, minoritized students showed a wider dispersion of purposes, compared to majoritized students. *Competence* was the most frequently mentioned purpose, particularly for majoritized students (> 87%) compared to minoritized students (64% of women and 76% of men). *Representation of identity* was more frequently mentioned by minoritized women (28%) than majoritized women (8%), and it was not mentioned by men at all. Representation included a drive to act as a role model for future generations:

I am Dominican-American and I have never met or heard of anyone studying or doing what I am doing. In the Hispanic culture, the science field is something that isn't brought up much and if it is it’s to become a doctor/nurse. I want to show younger Hispanic kids that they too can be a cool scientist! (Hispanic woman; chemistry and environmental science).

Students integrated both communal and agentic elements into their purpose, and many included multiple motivations that aided them in overcoming challenges:

When I started college I did not know what I do. I took many courses and found myself most interested in my math classes. Although it is a heavy workload, I enjoy math. It is like solving a puzzle. Sometimes it gets stressful and overwhelming if I fall behind or do not understand a topic. I try to focus on the positives to push through it. I think of how much I am learning, how many interesting applications there are for what I am learning, and I think of my future salary. (White woman; mathematics).

Further, communal purpose emerged differently across students. Some students emphasized direct collaboration and connection: “I hope to cherish those relationships I make through the workplace and clients. I think that a STEM pathway is a great way to make an impact in your career” (White woman; kinesiology). Other students emphasized more distal prosocial impact: “I would like to work on online products that have a high impact on people across the world. It is important to me to try and make an impact with my work, and there are a million different corners of software engineering that profoundly impact people's day to day lives, even if they do not realize it” (White man; computer science). The complexity and multiplicity of students’ purposes suggests it is important for them to articulate these for themselves.

### Summary

In sum, the content of students’ reflections revealed both similarities and differences across identity groups. As expected given rigorous STEM majors, most students wrote about coursework challenges and competence purposes. Even when students reported agentic and communal motives, how these motives crystallized within STEM varied from student to student. For students whose identities are well-represented in their majors, their challenges and purpose are fairly localized to their coursework and competence; for minoritized students, their challenges and purpose are more widely dispersed.

These student responses illustrate the double-edged sword of marginalized identities. For students from historically excluded groups, identity consideration emerged as both a stressor and purpose. Particularly for minoritized women, identity both led to challenges within their STEM majors and provided a source of meaning.

## Discussion

Across both race and gender, students from minoritized and majority groups who wrote briefly about *why* they were pursuing their STEM degree showed more beneficial cognitions and attitudes. They perceived greater opportunities to fulfill their agentic and communal goals, and they reported more enjoyment and certainty about their majors. Further, students who reflected on their purpose reported greater authentic belonging, as well as reduced stress appraisals. This robust purpose effect holds promise to elevate students, especially those who constantly must contend with questions of whether they “have it”—as individuals and as members of historically excluded groups.

These results are a warning call that the “same” STEM classroom is not psychologically equivalent to all students, and higher education has more work to do to reach equity in terms of inclusion and psychological safety. Racially minoritized students, and minoritized women especially, reported higher levels of stress and lower coping efficacy/resources. Because they started at a much higher level of stress, even a beneficial purpose reflection could not eliminate group disparities in stress. Even after considering purpose, minoritized women reported stress appraisals that were *still higher* than the those of majoritized men considering their challenges. These results are alarming, given the hazards of the Strong Black Woman stereotype^31^. A level playing field will not be achieved by the individual action of reflecting on purpose: Broader systemic changes are necessary to achieve equity.

This study illuminated plausible psychological processes for how reflecting on purpose realizes benefits. Students who reflected on their purpose perceived that their major allowed them greater opportunity to connect with or help others (communal affordances), and greater opportunity for self-advancement and achievement (agentic affordances). Each of these perceived opportunities in the major was distinctly associated with a sense that students could be their real selves and be accepted in their STEM major. The perception of agentic opportunities was distinctly associated with a reduced appraisal of stress. These findings add to the well-documented relationship between perceived control and stress^25^, but expand to include perceptions of one’s environment rather than oneself. Indeed, perceiving agentic opportunities in their major may provide students with greater ability to deploy their coping resources to manage their stressors. Delineating the unique role of agentic affordances in stress appraisals provides an important step for future research and intervention. All students experience stress along STEM pathways, and clearly providing direct paths to agentic opportunities in STEM environments—for all students—can mitigate stress appraisals.

These results provide insight about how particular motives and cognitions matter in STEM trajectories. Goal congruity theory^4,5^ posits that perceiving a greater opportunity to fulfill valued goals leads to motivational, affective, and cognitive benefits. The current findings advance this theoretical framework with more precise evidence about when particular affordances matter: Both agentic and communal affordances uniquely related to authentic belonging, but only agentic affordances related to reduced stress appraisals. Initiatives to bolster students’ communal opportunities in STEM deserve focus because STEM fields are perceived as deficient in these opportunities. Communal opportunities matter for authenticity and belonging, perhaps especially for students historically excluded from STEM^11,13^. Yet highlighting communal opportunities cannot come at the cost of highlighting agentic opportunities. For students pursuing STEM majors, agentic goals and affordances matter^32^. Classrooms, departments, and institutions that can clarify how their programs aid students in reaching *both* their communal and agentic goals are those that will recruit and retain students from a wide range of backgrounds.

The current research provides causal evidence of the benefits of a brief purpose reflection for a wide range of students, and future research can build on this foundation to understand when and how identity might moderate the accessibility or impact of purpose reflection. For example, identities may shape outcomes at different parts of the developmental trajectory: Articulating purpose might matter more for historically excluded groups at key decision points, such as choosing a major or applying for jobs. Further, future research can investigate the consequences of having one’s purpose reflected or dismissed by the local culture. STEM faculty who are from majoritized identities may be less likely to know or ask about minoritized students’ purposes—even though these are the students who might gain the most from such purpose integration in STEM fields. We acknowledge that the current sample did not include gender nonbinary and other gender-identified individuals; yet, we hope that this initial empirical work provides a foundation to investigate a range of other minoritized and marginalized identities to understand if purpose benefits emerge similarly in content and in impact.

The randomized, controlled nature of this experiment provides causal evidence that purpose reflection can benefit students. We note that students articulated their purpose, and thus it is not the instructors’ purview to tell students what their purpose is but to create space for reflection. As such, reflections might be incorporated into instruction, advising, and mentoring in straightforward ways. For example, STEM faculty, even in large lecture courses, might ask students to write briefly about their purpose as an attendance check or extra credit. Advisors and mentors could prompt students to articulate their “why” and consider exploring paths that make these purposes more concrete in their day-to-day lives. And students, on their own, might take a moment to consider what is most important to them about this pathway. Pursuing a STEM major is challenging—that is the nature of the work. Yet, connecting students to their “why” can help move them forward with meaning, for the ultimate benefit of both their own lives and building a vibrant, diverse, and innovative STEM workforce.

## Method

Procedures and materials were approved by Indiana University’s Institutional Review Board under protocol number #11661. The experiment was performed in accordance with relevant guidelines and regulations. Participants engaged in the study anonymously, and participant data was stored securely. Informed consent was obtained from all participants.

### Participants

The study recruited undergraduate students in engineering or physical sciences majors from Prolific. Of 575 initial respondents, 82 respondents were omitted because they did not complete more than 50% of the survey and 3 respondents were omitted because they did not meet the study criterion of a STEM major or minor. Data from 24 respondents who did not identify gender as male or female were also omitted, given insufficient data to analyze nonbinary and other gender-identified respondents as a group; we recognize this as a limitation.

The resulting sample was 466 participants (232 women and 234 men), including 280 White students (60.1%), 134 Latino(a/x) students (28.8%), 93 Black/African American students (20%), 11 Asian/Asian American students (2.4%), and 10 Native American/Indigenous students (2.1%). Participants could select multiple racial and ethnic identity options. For analyses, Black/African-American, Latinx, and Native American/Indigenous students were categorized as minoritized students, and White and/or Asian students were categorized as majoritized students given current representation in STEM fields^1^.

Participants were predominantly physical science majors (*n* = 225) and engineering majors (*n* = 175); other majors included technology (*n* = 66) and math (*n* = 57), and life sciences (*n* = 38). One student reported a STEM minor. Participants indicated multiple majors if applicable. Most participants were in their first four years of higher education (18.2% first year; 24.2% second year; 28.1% third year; 24.7% fourth year, 3.0% fifth year or second bachelor’s/similar degree; 1.7% first year masters or similar).

### Power analysis

For a 2 (Reflection Condition) × 2 (Gender) × 2 (Majoritized or Minoritized Race/Ethnicity) between-subjects ANOVA, post hoc sensitivity analysis^29,30^ indicated a detectable effect size of *f* = 0.130 (power = 0.80; α = 0.05, *n* = 466). A target sample size of 500 (250 minoritized students) was determined using G*Power 3.0 before data collection; we closed data collection when the pool of minoritized students majoring in engineering and physical sciences was exhausted on the recruitment platform.

### Procedure

After providing informed consent, participants were randomly assigned to one of two reflection conditions. Half of the participants were asked to reflect on their challenges, and half of the participants were asked to reflect on their challenges and integrate their purpose into considering these difficulties. In the challenges condition, participants were prompted with the following:

College can be a difficult time because you are asked to learn new things and figure out your next steps. In the next few minutes, use this space to think about these challenges. What are the challenges associated with your training? How do these show up in your everyday life?

In the purpose condition, participants were prompted with the following:

College can be a difficult time because you are asked to learn new things and figure out your next steps. But often these difficulties are worth it if we are doing something that matters. In the next few minutes, use this space to think about these challenges and why you decided to be a STEM major. What is it you hope to do with your training? What is important to you about this pathway?

Participants were given 5 min to write about their randomly-assigned prompt.

### Measures

#### Communal and agentic affordances of STEM major

Participants rated how much their major fulfilled communal and agentic goals on scales ranging from 1 (*Not at all*) to 7 (*Extremely*). Students rated how much their *major* provided opportunities to fulfill 6 communal goals (*work with or collaborate with others, learn material that benefits others, form connections with others, increase your affiliation with others, be altruistic*; *help others;* α = 0.88) and 6 agentic goals *(gain competence; gain new skills; gain a deeper understanding of course materials; gain success; be independent; demonstrate your skills;* α = 0.85). Items were averaged within subscale. Affordances scales were drawn from previous work showing high reliability and convergent validity^33^.

#### Attitudes about major

On scales ranging from 1 (Strongly Disagree) to 7 (*Strongly Agree*), participants rated how much they *enjoy* the courses and activities for their major and how often they think about changing their major (*certainty)*^22^.

#### Authentic belonging

Participants rated 7 items related to authentic belonging in their major. Five items were drawn from previous research^22,34^: *I feel like I belong in my major, I feel like I fit in with the people in my major, I feel comfortable around the people in my major, I feel included by people in my major, I feel like I can be myself with the people in my major)*. We added two face-valid items consistent with a workplace authenticity measure^35^ to increase internal consistency (*I feel like my authentic self when I’m with the people in my major, I feel like I am my true self with the people in my major).* Together, the 7 items showed high internal consistency (α = 0.95*)*. Items were rated on scales ranging from 1 (*Strongly Disagree*) to 7 (*Strongly Agree*) and averaged.

#### Attitudes and beliefs about the expected career

Participants completed parallel items focused on their anticipated careers: *Communal affordances* (α = 0.88), *agentic affordances (*α = 0.82), *enjoyment*, *certainty*, and *authentic belonging* (α = 0.96).

#### Stress appraisal

Participants rated one item assessing demand (i.e., how much stress they experience on a day-to-day basis in their STEM courses), and two items assessing capacity (how confident they feel that they can handle the stress; how much they feel that they have the resources and support to handle the stress). Each item was rated on a 7-point scale ranging from *not at all* to *an extreme amount/extremely.* Following common practice in the stress appraisal literature^36,37^, we computed an index of stress appraisal reflecting the participant’s appraisal of demands relative to capacity. This discrepancy score subtracted coping capacity [average of confidence and resources, *r*(463) = 0.512, *p* < 0.001] from perceived stress. Larger positive scores reflect that stress surpasses capacity, whereas larger negative scores reflect that capacity surpasses stress.

### Thematic coding of reflection content

Using a thematic coding protocol, each written response was coded by two trained independent coders for the presence of several constructs drawn from previous literature^4,13,38^. Brief descriptions of each construct were provided to each coder. Coders trained on a subset of responses and received feedback before coding the data. Coders were blind to participants’ race/ethnicity and gender (unless mentioned by the participant). Overall, coders reached high agreement (93.7%) on the presence or absence of each construct. Disagreements were resolved by a member of the co-author team.

The purpose codes included both communal and agentic motives to pursue STEM (see example excerpts in Table 4). Communal motives included *connection* motives to work alongside others, *prosocial* motives to benefit others, and *representation* motives to succeed in STEM as a role model for other minoritized students. Agentic motives included *competence* motives focused on demonstrating skills and learning about STEM topics, *financial* motives involving pursuing monetary rewards and opportunities, and *status* motives involving a desire to obtain recognition and power over others.

**Table 4:** Sample excerpts of coded motivational themes.

Thematic construct	Sample excerpt
Communal motives	
Connection	“What's important to me about this pathway is the collaboration of different minds/perspectives to solve a problem and I value that in a career”
Prosocial	“I hope that being a system admin after college will ensure that online users' safety and privacy are guaranteed… It is essential to be in STEM/IT as everyone deserves to feel safe on the web”
Representation	“I hope within my time as a math major I have paved more of an open and understanding pathway for future women in math classes at the collegiate level”
Agentic motives	
Competence	“I also remind myself why I wanted to study physics in the first place—I have a curious mind and want to better understand how the world/universe operates”
Financial	“Frankly, I chose a STEM major because I know it would make me money”
Status	“To me, electrical engineering is important since I can use it as a means of making my impact on the world. I believe that through technology I can have more influence on the things around me and I can do my part to advance society”

The challenge codes included six themes (see Table 5 for excerpts). *Coursework difficulty* included the struggles associated with completing STEM coursework. *Social stressor* challenges included difficulties reaching out for help or building and maintaining relationships. *Work and family* challenges accounted for stressors associated with managing roles beyond the STEM student role. The *resources* theme captured difficulties in obtaining structural resources and meeting basic needs, such as housing or food. *Uncertainty* captured feelings of stress related to deciding one’s career path or college major. Finally, *identity stressors* referred to negative stereotypes and hostile environments students experienced due to minoritized gender, racial, or other social identities.

**Table 5:** Sample excerpts of coded challenge themes.

Thematic construct	Sample excerpt
Coursework	“I feel like one of my challenges is having to learn a lot of complex material in a short period of time. This shows up in my daily life because I am constantly stressed and busy trying to keep up with school”
Social stressor	“I often felt isolated from my peers in my courses due to my educational background… I often felt that I was not able to spend as much time with friends as others who were smarter than me”
Work and family	“Computer Science requires a ton of math so it is a priority. I have a fiancé and a 1 year old son, so this definitely causes problems at home with how much time I’m not putting into family time. Although it’s for the good of the family, college takes a lot of time away from personal things”
Resources	“I have faced many challenges in school and restarted later in life than a lot of people. Money has been an issue and I had to stop and restart once again because I got pregnant and needed to work full time to prepare for my child”
Uncertainty	“I currently feel like I'm facing a huge challenge in trying to decide what I want out of life after college. I feel an enormous amount of pressure to decide soon and create a vision of what I'm working towards, especially since I'm paying so much to get there. The idea of having to switch majors and possibly have to pay for more terrifies me”
Identity stressor	“Being a female in a STEM program is definitely hard to identify with professors and other students, it is difficult to find camaraderie in the program”

### Supplementary Information

Supplementary Information.

## Data Availability

The data used and/or analyzed for the current study are available from the corresponding author upon reasonable request.
