# Supplementary material for: Purpose reflection benefits minoritized students’ motivation and well-being in STEM
Source: Sci Rep. 2024 Jan 3;14:466. doi: 10.1038/s41598-023-50302-1 (PMC10764869; doi:10.1038/s41598-023-50302-1)
Supplement: Supplementary file 1 — Supplementary Information. [file 41598_2023_50302_MOESM1_ESM.docx]

**Supplementary Materials for**

***Purpose Reflection Benefits Minoritized Students’ Motivation and Well-Being in STEM***

**Effects of Purpose Reflection on Individual Stress and Coping Items**

Each component of the stress appraisal was influenced by purpose reflection: Students who reflected on purpose reported lower stress, *F*(1, 458) = 13.055, *p* < .001, *η_p_^2^* = .028, greater confidence to handle stress, *F*(1, 458) = 8.921, *p* = .003, *η_p_^2^* = .019, and greater resources to handle stress, *F*(1, 457) = 9.918, *p* = .002, *η_p_^2^* = .021.

**Effects of Race and Gender**

This supplement reports the significant effects of race and gender that do not interact with the reflection condition effects reported in the main text. All analyses were conducted in 2 (Reflection Condition) × 2 (Gender) × 2 (Majoritized or Minoritized Race/Ethnicity) between-subjects analyses of variance (ANOVAs). Full statistics are available from the authors upon request.

Students from historically excluded groups reported more negative cognitions and attitudes. Women, compared to men, reported fewer beneficial cognitions about their major (higher certainty, enjoyment) and less positive well-being (major and career authentic belonging, lower stress, greater efficacy to handle stress, and greater resources and support); *p*s < .05 for all main effects of gender. Racially minoritized students, compared to racially majoritized students, reported fewer beneficial cognitions about their major (authentic belonging, certainty), less certainty about their career, and less efficacy to handle stress, *p*s < .05 for all main effects of race.

Additionally, a two-way interaction between gender and race emerged for affordances. The Gender × Race interaction, *F*(1, 458) = 3.88, *p* = .049, *η_p_^2^* = .008, reflected that women’s tendency to perceive fewer goal opportunities was larger among racially minoritized students: A larger gender gap emerged emerged for minoritized students, *F*(1, 458) = 3.389, *p* = .066, *η_p_^2^* = .007, Cohen’s *d* = .21, than for majoritized students *F*(1, 458) = .873, *p* = .351, *η_p_^2^* = .002, *d* = 0.09.

Stress appraisals (i.e., discrepancy scores indexing whether stress outweighs coping) also showed robust variation across gender and race. Greater stress appraisals emerged for women than men, *F*(1, 458) = 25.40, *p* < .001, *η_p_^2^* = .053, and for minoritized than majoritized students, albeit marginally, *F*(1, 458) = 3.42, *p* = .065, *η_p_^2^* = .007. Further, stress appraisals were acutely high for racially minoritized women, as reflected in the Gender × Race interaction, *F*(1, 458) = 4.60, *p* = .033, *η_p_^2^* = .010. Minoritized women showed significantly higher stress appraisals than did minoritized men, *F*(1, 458) = 25.21, *p* < .001, *η_p_^2^* = .052, *d* = .60; this gender difference was smaller among majoritized students, *F*(1, 458) = 4.293, *p* = .039, *η_p_^2^* = .009, *d* = .31.

In sum, the race and gender variations that emerged across measures converged to show a more challenging context for students who identify as women, as Black/Latinx/Native, or both.
